# Implementation of earlier antibiotic administration in patients with severe sepsis and septic shock in Japan: a descriptive analysis of a prospective observational study

**DOI:** 10.1186/s13054-019-2644-x

**Published:** 2019-11-19

**Authors:** Toshikazu Abe, Shigeki Kushimoto, Yasuharu Tokuda, Gary S. Phillips, Andrew Rhodes, Takehiro Sugiyama, Akira Komori, Hiroki Iriyama, Hiroshi Ogura, Seitaro Fujishima, Atsushi Shiraishi, Daizoh Saitoh, Toshihiko Mayumi, Toshio Naito, Kiyotsugu Takuma, Taka-aki Nakada, Yasukazu Shiino, Takehiko Tarui, Toru Hifumi, Yasuhiro Otomo, Kohji Okamoto, Yutaka Umemura, Joji Kotani, Yuichiro Sakamoto, Junichi Sasaki, Shin-ichiro Shiraishi, Ryosuke Tsuruta, Akiyoshi Hagiwara, Kazuma Yamakawa, Tomohiko Masuno, Naoshi Takeyama, Norio Yamashita, Hiroto Ikeda, Masashi Ueyama, Satoshi Gando, on behalf of JAAM FORECAST group, Osamu Tasaki, Yasumitsu Mizobata, Hiraku Funakoshi, Toshiro Okuyama, Iwao Yamashita, Toshio Kanai, Yasuo Yamada, Mayuki Aibiki, Keiji Sato, Susumu Yamashita, Kenichi Yoshida, Shunji Kasaoka, Akihide Kon, Hiroshi Rinka, Hiroshi Kato, Hiroshi Okudera, Eichi Narimatsu, Toshifumi Fujiwara, Manabu Sugita, Yasuo Shichinohe, Hajime Nakae, Ryouji Iiduka, Mitsunobu Nakamura, Yuji Murata, Yoshitake Sato, Hiroyasu Ishikura, Yasuhiro Myojo, Yasuyuki Tsujita, Kosaku Kinoshita, Hiroyuki Yamaguchi, Toshihiro Sakurai, Satoru Miyatake, Takao Saotome, Susumu Yasuda, Toshikazu Abe, Hiroshi Ogura, Yutaka Umemura, Atsushi Shiraishi, Shigeki Kushimoto, Daizoh Saitoh, Seitaro Fujishima, Junichi Sasaki, Toshihiko Mayumi, Yasukazu Shiino, Taka-aki Nakada, Takehiko Tarui, Toru Hifumi, Yasuhiro Otomo, Joji Kotani, Yuichiro Sakamoto, Shin-ichiro Shiraishi, Kiyotsugu Takuma, Ryosuke Tsuruta, Akiyoshi Hagiwara, Kazuma Yamakawa, Naoshi Takeyama, Norio Yamashita, Hiroto Ikeda, Yasuaki Mizushima, Satoshi Gando

**Affiliations:** 1 Department of General Medicine, Juntendo University, Tokyo, Japan; 2 Health Services Research and Development Center, University of Tsukuba, Tsukuba, Japan; 3 Department of Health Services Research, Faculty of Medicine, University of Tsukuba, Tsukuba, Japan; 4 Division of Emergency and Critical Care Medicine, Tohoku University Graduate School of Medicine, Sendai, Japan; 5 Department of Medicine, Muribushi Project for Okinawa Residency Programs, Urasoe, Japan; 6 Department of Biomedical Informatics, Ohio State University, Columbus, OH USA; 7 Department of Intensive Care Medicine, St George’s University Hospitals Foundation Trust, London, UK; 8 Diabetes and Metabolism Information Center, Research Institute, National Center for Global Health and Medicine, Tokyo, Japan; 9 Department of Public Health/Health Policy, Graduate School of Medicine, The University of Tokyo, Tokyo, Japan; 10 Department of Traumatology and Acute Critical Medicine, Osaka University Graduate School of Medicine, Osaka, Japan; 11 Center for General Medicine Education, Keio University School of Medicine, Tokyo, Japan; 12 Emergency and Trauma Center, Kameda Medical Center, Kamogawa, Japan; 13 Division of Traumatology, Research Institute, National Defense Medical College, Tokorozawa, Japan; 14 Department of Emergency Medicine, School of Medicine, University of Occupational and Environmental Health, Kitakyushu, Japan; 15 Emergency & Critical Care Center, Kawasaki Municipal Kawasaki Hospital, Kawasaki, Japan; 16 Department of Emergency and Critical Care Medicine, Chiba University Graduate School of Medicine, Chiba, Japan; 17 Department of Acute Medicine, Kawasaki Medical School, Kurashiki, Japan; 18 Department of Trauma and Critical Care Medicine, Kyorin University School of Medicine, Tokyo, Japan; 19 Department of Emergency and Critical Care Medicine, St. Luke’s International Hospital, Tokyo, Japan; 20 Trauma and Acute Critical Care Center, Medical Hospital, Tokyo Medical and Dental University, Tokyo, Japan; 21 Department of Surgery, Center for Gastroenterology and Liver Disease, Kitakyushu City Yahata Hospital, Kitakyushu, Japan; 22 Department of Disaster and Emergency Medicine, Kobe University Graduate School of Medicine, Kobe, Japan; 23 Emergency and Critical Care Medicine, Saga University Hospital, Saga, Japan; 24 Department of Emergency and Critical Care Medicine, Keio University School of Medicine, Tokyo, Japan; 25 Department of Emergency and Critical Care Medicine, Aizu Chuo Hospital, Aizuwakamatsu, Japan; 26 Advanced Medical Emergency & Critical Care Center, Yamaguchi University Hospital, Ube, Japan; 27 Department of Emergency Medicine, Niizashiki Chuo General Hospital, Niiza, Japan; 28 Division of Trauma and Surgical Critical Care, Osaka General Medical Center, Osaka, Japan; 29 Department of Emergency and Critical Care Medicine, Nippon Medical School, Tokyo, Japan; 30 Advanced Critical Care Center, Aichi Medical University Hospital, Nagakute, Japan; 31 Advanced Emergency Medical Service Center, Kurume University Hospital, Kurume, Japan; 32 Department of Emergency Medicine, Teikyo University School of Medicine, Tokyo, Japan; 33 Department of Trauma, Critical Care Medicine, and Burn Center, Japan Community Healthcare Organization, Chukyo Hospital, Nagoya, Japan; 34 Division of Acute and Critical Care Medicine, Hokkaido University Graduate School of Medicine, Sapporo, Japan; 35 Department of Acute and Critical Care Medicine, Sapporo Higashi Tokushukai Hospital, Sapporo, Japan

**Keywords:** Sepsis, Antibiotic, Bundle, Protocols

## Abstract

**Background:**

Time to antibiotic administration is a key element in sepsis care; however, it is difficult to implement sepsis care bundles. Additionally, sepsis is different from other emergent conditions including acute coronary syndrome, stroke, or trauma. We aimed to describe the association between time to antibiotic administration and outcomes in patients with severe sepsis and septic shock in Japan.

**Methods:**

This prospective observational study enrolled 1184 adult patients diagnosed with severe sepsis based on the Sepsis-2 criteria and admitted to 59 intensive care units (ICUs) in Japan between January 1, 2016, and March 31, 2017, as the sepsis cohort of the Focused Outcomes Research in Emergency Care in Acute Respiratory Distress Syndrome, Sepsis and Trauma (FORECAST) study. We compared the characteristics and in-hospital mortality of patients administered with antibiotics at varying durations after sepsis recognition, i.e., 0–60, 61–120, 121–180, 181–240, 241–360, and 361–1440 min, and estimated the impact of antibiotic timing on risk-adjusted in-hospital mortality using the generalized estimating equation model (GEE) with an exchangeable, within-group correlation matrix, with “hospital” as the grouping variable.

**Results:**

Data from 1124 patients in 54 hospitals were used for analyses. Of these, 30.5% and 73.9% received antibiotics within 1 h and 3 h, respectively. Overall, the median time to antibiotic administration was 102 min [interquartile range (IQR), 55–189]. Compared with patients diagnosed in the emergency department [90 min (IQR, 48–164 min)], time to antibiotic administration was shortest in patients diagnosed in ICUs [60 min (39–180 min)] and longest in patients transferred from wards [120 min (62–226)]. Overall crude mortality was 23.4%, where patients in the 0–60 min group had the highest mortality (28.0%) and a risk-adjusted mortality rate [28.7% (95% CI 23.3–34.1%)], whereas those in the 61–120 min group had the lowest mortality (20.2%) and risk-adjusted mortality rates [21.6% (95% CI 16.5–26.6%)]. Differences in mortality were noted only between the 0–60 min and 61–120 min groups.

**Conclusions:**

We could not find any association between earlier antibiotic administration and reduction in in-hospital mortality in patients with severe sepsis.

## Key points

- In Japan, one third of the patients received antibiotics within 1 h and three fourths within 3 h of sepsis recognition.
- Our descriptive results do not support early antibiotic administration, i.e., within 1 h after diagnosis, for reducing in-hospital mortality in patients with severe sepsis and septic shock.

## Background

Time to antibiotic administration is a key element in sepsis care, and the Surviving Sepsis Campaign (SSC) guidelines (2004, 2008, 2012, and 2016) have repeatedly recommended initiating empirical broad-spectrum therapy within 3 h from triage or sepsis recognition [1–4]. However, the updated 2018 SSC guidelines recommend a 1-h window for antibiotic administration following the recognition of sepsis as a reasonable approach [5]; this update has been significantly debated, and it remains controversial [6]. Although it would be impossible to argue against appropriate and timely antibiotic therapy for sepsis considering its time-sensitive nature, sepsis is different from other emergent conditions such as acute coronary syndrome, stroke, or trauma. Specifically, its recognition by healthcare providers within 1 h of presentation may be difficult to achieve in real-life settings because of vague presenting symptoms and the fact that its exact onset is mostly unobservable. Further, the only randomized controlled trial (RCT) that evaluated early antibiotic use in patients with suspected infection failed to reduce mortality, although this was in the pre-hospital setting [7].

We prospectively evaluated the characteristics and management of patients with severe sepsis in the Focused Outcomes Research in Emergency Care in Acute Respiratory Distress Syndrome, Sepsis, and Trauma (FORECAST) study in Japan [8]. We then used the FORECAST database to describe the association between time to antibiotic administration and outcomes in patients with severe sepsis and septic shock treated in real-world clinical settings.

## Methods

The study protocol was reviewed and approved by the ethics committee of all participating institutions in the Japanese Association for Acute Medicine (JAAM) study group, Japan (IRB No.014-0306 on Hokkaido University, a representative institution for FORECAST). Obtaining an informed consent from a study participant was waived under the approval of the ethics committees.

### Design and setting

This is a predefined secondary analysis of the FORECAST study. We predefined the secondary analyses when we set variables of the FORECAST study. It selected a cohort of patients with severe sepsis and septic shock who were registered in the FORECAST study, which was a multi-center, prospective data collection study on acutely ill patients that included those with acute respiratory distress syndrome, sepsis, and trauma. The FORECAST study obtained data from patients admitted to 59 intensive care units (ICUs) in Japan and was conducted from January 1, 2016, to March 31, 2017.

### Participants

We included adult patients (≥ 16 years) diagnosed with severe sepsis and septic shock based on the Sepsis-2 criteria published in 2003 [9] and admitted to the ICU. The exclusion criteria were unfavorable sustained life-care or post-cardiopulmonary arrest resuscitation status at the time of sepsis recognition, missing data on antibiotic timing or in-hospital mortality, or time to antibiotic use > 1440 min.

### Data collection

Relevant patient data, originally compiled by the FORECAST investigators, were obtained from the FORECAST database. Hospital information obtained included number, specialty, type of facility and staff, number of patients, and number of beds. Patient data, collected as part of the clinical workup, included demographic characteristics of patients, organ dysfunctions, sepsis-related severity scores, time to antibiotic administration, in-hospital mortality, 28-day mortality, ICU-free days, ventilator-free days (VFD), and length of hospital stay. Additionally, we obtained data on compliance with established sepsis care bundles, such as measurement of the initial serum lactate levels within 3 h.

### Data definitions

Sepsis care bundles were defined according to the SSC guidelines (2012) [3] as to whether all bundle elements were achieved within the appropriate time frame (i.e., 3 or 6 h) and if they adhered to indications (i.e., septic shock or lactate level > 4 mmol/L). For all patients, protocol initiation time was defined as the time of sepsis recognition at the emergency department (ED), ward, or ICU. Sepsis recognition was a clinical judgment, wherein a physician-in-charge had suspected sepsis at the initial evaluation. Timestamp was recorded in the database by a physician-in-charge. Patients with antibiotic before arrival were recognized as patients with infection but not sepsis. After arrival, time would start when a physician-in-charge recognized a patient with sepsis. Time from sepsis recognition to initiation of antibiotics (time to antibiotic) was divided into six groups defined as 0–60, 61–120, 121–180, 181–240, 241–360, and 361–1440 min. Septic shock was defined based on the Sepsis-2 criteria [9], and VFD was defined as the number of days within the first 28 days after hospital admission during which a patient was able to breathe without a ventilator. VFD for patients who died during the study period was listed as 0. ICU-free days were similarly calculated.

### Analysis

The primary outcome was in-hospital mortality. Secondary outcomes were VFD and ICU-free days. The exposure was time to antibiotic. Because the missing data were low, no assumptions were made for this factor.

Descriptive statistics included frequency and percentage for categorical variables and mean ± standard deviation (SD) or medians and interquartile range (IQR) for continuous variables, as appropriate. We compared baseline characteristics and outcomes among patients with severe sepsis in the six time to antibiotic therapy groups (0–60, 61–120, 121–180, 181–240, 241–360, and 361–1440 min) using analysis of variance, Kruskal-Wallis test or chi-square test, as required.

The impact of antibiotic timing on risk-adjusted hospital mortality was estimated using the generalized estimating equation (GEE) model with an exchangeable within-group correlation matrix where the hospital was the panel or grouping variable. The following covariates were specified a priori based on clinical experience and prior studies: patient age, gender, admission source (ED, ward, or in ICU), Charlson comorbidity index (CCI), antibiotic use before arrival, site of infection (e.g., lung, abdomen, urinary tract, soft tissue, central nerve system, or blood stream-related), sepsis-related organ failure assessment (SOFA) score, and intravenous fluid bolus completed within 3 h (30 mg/kg crystalloid). We also performed the same analysis after replacing time to antibiotic as a continuous variable. In a subgroup analysis, we stratified patients with septic shock and those from only ED based on hospital admission source and analyzed these subgroups as described in the primary analysis [i.e., the GEE model adjusted patient age, gender, CCI, antibiotic use before arrival, site of infection, SOFA score, and intravenous fluid bolus completed within 3 h (30 mg/kg crystalloid)].

All statistical analyses were performed using Stata software version 15.1 (StataCorp, TX, USA).

## Results

We recruited 1184 patients with severe sepsis who were admitted to ICUs at participating institutions during the FORECAST study period. Of these, 60 patients were excluded because of missing data on the timing of antibiotic administration (*n* = 11) or in-hospital mortality (*n* = 33) or because the time to antibiotic was greater than 1440 min. Of the remaining 1124 participants who were admitted to 54 hospitals, 30.5% of the patients received antibiotics within 1 h, and 73.9% of the patients received antibiotics within 3 h. Overall, the median time to antibiotic administration was 102 min (IQR, 55–189), and compared with patients diagnosed in the ED [90 min (IQR, 48–164 min)], time to antibiotic administration was shortest in patients diagnosed in ICUs [60 min (39–180 min)] and longest in patients who had been transferred from wards [120 min (62–226 min)], implying that patients diagnosed in the ED or the ICU received antibiotics earlier than those in the ward.

Baseline characteristics, categorized based on the timing of antibiotic administration, are detailed in Table 1. The 0–60, 61–120, and 121–180 min groups received lower pre-antibiotic therapy, such as oral medicines for infection, than the other groups. The 0–60, 61–120, and 121–180 min groups were also more likely to achieve the 3-h bundle, such as obtaining blood cultures and intravenous fluid bolus than the other groups.

**Table 1:** Baseline characteristics of patients with severe sepsis and septic shock according to the timing of antibiotic administration

		Time to antibiotic use (min)						*p* value
		0–60	61–120	121–180	181–240	241–360	361–1440	
Number of patients, *n*	1124	343	327	161	94	103	96	
Age at admission in years, median (IQR)		73 (65–82)	73 (63–81)	75 (66–82)	68 (58–78)	74 (67–81)	74 (63–83)	0.035
Gender, *n* (% male)		196 (57.1)	190 (58.1)	102 (63.4)	65 (69.2)	65 (63.1)	57 (59.4)	0.295
Admission source, *n* (%)	ED	210 (61.4)	194 (59.3)	94 (58.4)	45 (47.9)	56 (54.4)	32 (33.3)	< 0.01
	ICU	22 (6.4)	8 (2.5)	3 (1.9)	3 (3.2)	3 (2.9)	4 (4.2)	
	Ward	110 (32.2)	125 (38.2)	64 (39.8)	46 (48.9)	44 (42.7)	60 (62.5)	
Carlson comorbidity index, median (IQR)		1 (0–2)	1 (0–2)	1 (0–2)	1 (0–2)	1 (0–2)	1 (0–2)	0.8543
Antibiotic use for infection before arrival, *n* (%)		112 (32.8)	100 (30.8)	51 (31.9)	41 (44.1)	42 (41.6)	55 (57.3)	< 0.01
Suspected site of infection, *n* (%)	Lung	91 (26.5)	102 (31.2)	62 (38.5)	31 (33.0)	34 (33.0)	31 (32.2)	0.003
	Abdomen	102 (29.7)	69 (21.1)	34 (21.1)	22 (23.4)	30 (29.1)	32 (33.3)	
	Urinary tract	73 (21.3)	76 (23.2)	27 (16.8)	13 (13.8)	12 (11.7)	11 (11.5)	
	Soft tissue and wound	32 (9.3)	44 (13.5)	16 (9.9)	15 (16.0)	12 (11.7)	5 (5.2)	
	Central nervous system	8 (2.3)	2 (0.6)	3 (1.9)	4 (4.3)	4 (3.9)	0 (0)	
	Blood stream-related	12 (3.5)	18 (5.5)	10 (6.2)	7 (7.5)	6 (5.8)	10 (10.4)	
	Others	25 (7.3)	16 (4.9)	9 (5.6)	2 (2.1)	5 (4.9)	7 (7.3)	
Positivity of blood cultures, *n* (%)		192 (56.3)	162 (49.7)	86 (54.1)	50 (53.2)	60 (58.3)	52 (54.2)	0.561
Septic shock, *n* (%)		215 (62.7)	207 (63.3)	94 (58.4)	61 (64.9)	69 (67.0)	57 (59.4)	0.735
Mechanical ventilation, *n* (%)		142 (42.3)	129 (40.6)	58 (37.2)	36 (39.1)	43 (42.2)	38 (40.9)	0.932
Organ dysfunction on arrival	Hypotension, *n* (%)	193 (56.3)	178 (54.4)	74 (46.0)	59 (62.8)	65 (63.1)	46 (47.9)	0.029
	Hyperlactatemia (> 2 mmol/L), *n* (%)	240 (70.0)	214 (65.4)	119 (73.9)	61 (64.9)	69 (67.0)	45 (46.9)	< 0.01
	Acute kidney injury (creatinine > 2 mg/dL), *n* (%)	134 (39.1)	118 (36.1)	64 (39.8)	34 (36.2)	37 (35.9)	37 (38.5)	0.946
	Acute lung injury, *n* (%)	115 (33.5)	120 (36.7)	72 (44.7)	37 (39.4)	36 (35.0)	37 (38.5)	0.269
	Hyperbilirubinemia (> 2.0 mg/dL), *n* (%)	58 (16.9)	48 (14.7)	31 (19.3)	17 (18.1)	17 (16.5)	21 (21.9)	0.62
	Thrombocytopenia (< 100,000/μL), *n* (%)	104 (30.3)	91 (27.8)	43 (26.7)	29 (30.9)	31 (30.1)	28 (29.2)	0.951
	Coagulopathy (INR > 1.5), *n* (%)	70 (20.4)	61 (18.7)	25 (15.5)	18 (19.2)	15 (14.6)	18 (18.8)	0.72
Number of organ dysfunction, median (IQR)		2 (2–4)	2 (1–3)	2 (2–3)	3 (2–4)	2 (1–3)	2 (1–3)	0.1893
APACHE II score, median (IQR)		22 (17–30)	22 (16–28)	23 (18–31)	21 (16–29)	25 (19–32)	24 (18–29)	0.1613
SOFA score, median (IQR)		9 (6–11)	8 (6–11)	9 (5–11)	8 (6–11)	9 (6–12)	8 (5–11)	0.7155
Blood cultures before antibiotics completed within 3 h, *n* (%)		318 (93.0)	310 (95.4)	148 (91.9)	83 (88.3)	94 (91.3)	78 (81.3)	< 0.01
Intravenous fluid bolus completed within 3 h (30 mg/kg crystalloid), *n* (%)	No	37 (10.9)	50 (15.3)	32 (20.1)	21 (22.6)	22 (21.4)	27 (28.1)	0.001
	Yes	212 (62.4)	185 (56.6)	75 (47.2)	43 (46.2)	53 (51.5)	40 (41.7)	
	Not indicated	91 (26.8)	92 (28.1)	52 (32.7)	29 (31.2)	28 (27.2)	29 (30.2)	
Serum lactate obtained, *n* (%)		331 (96.8)	321 (98.2)	153 (95.0)	92 (97.9)	99 (96.1)	92 (95.8)	0.483
Entire 3-h bundle if indicated (*n* = 796), *n*/total (%)		194/249 (77.9)	170/234 (72.7)	67/107 (62.6)	25/64 (39.1)	33/75 (44.0)	21/67 (31.3)	< 0.01

Missing data: admission source = 1, antibiotic use before arrival = 8, blood cultures = 5, mechanical ventilation = 27, APACHE II score = 122, SOFA score = 171, blood cultures before antibiotics completed within 3 h = 3, intravenous fluid bolus completed within 3 h (30 mg/kg crystalloid) = 6, serum lactate obtained = 1

*ED* emergency department, *ICU* intensive care unit, *PT-INR* prothrombin time to international normalized ratio, *APACHE II* Acute Physiology and Chronic Health Evaluation II, SOFA sepsis-related organ failure assessment

**Fig. 1:**
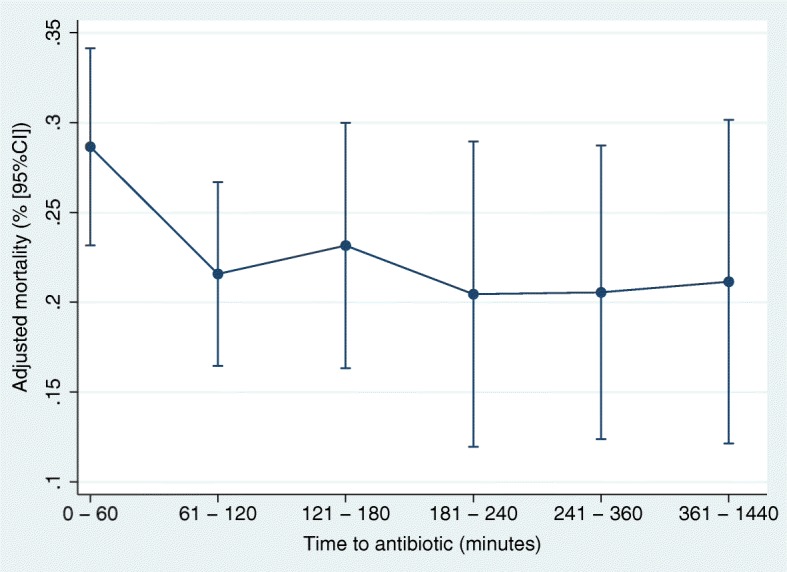
Adjusted in-hospital mortality according to the timing of antibiotic administration among patients with severe sepsis and septic shock. Adjusted by age, gender, admission source (emergency department, ward, or in intensive care unit), CCI, antibiotic use before arrival, site of infection (e.g., lung, abdomen, urinary tract, soft tissue, central nerve system, or blood stream-related), SOFA score, and intravenous fluid bolus completed within 3 h (30 mg/kg crystalloid)

Overall crude mortality was 23.4% in the study population. Crude mortalities were 27.7% and 16.2% among patients with and without shock, respectively. Patients received antibiotics within the first 60 min had the highest rates of mortality (28.0%) and risk-adjusted mortality [28.7% (95% CI 23.3–34.1); Table 2, Fig. 1], whereas that of patients administered with antibiotics between 61 and 120 min had the lowest (20.2%) and risk-adjusted mortality rates [21.6% (95% CI 16.5–26.6)]. Importantly, mortality was different only between patients in the 0–60 and 61–120 min groups. In addition, time to antibiotics as a continuous variable was not related to mortality, either (odds ratio 0.999 [0.997–1.000; *P* = 0.152]). Subgroup analysis yielded a crude mortality rate of 27.7% in patients with septic shock, and patients in the 0–60 min group continued to have the highest mortality (31.6%) and risk-adjusted mortality rates [30.0% (95% CI 23.5–36.5)], whereas those patients administered with antibiotics between 361 and 1440 min were the lowest [21.6% (95% CI 11.1–32.0)]. In addition, the subgroup analysis yielded a crude mortality rate of 21.9% in the ED patients, and patients in the 0–60 min group continued to have the highest mortality (29.1%) and risk-adjusted mortality rates (27.9% [95% CI 21.1–34.6]), whereas that in patients administered with antibiotics between 361 and 1440 min was the lowest [12.8% (95% CI 5.7–25.0)].

**Table 2:** Outcomes of patients with severe sepsis and septic shock according to the time to antibiotics

	Time to antibiotic use (min)						*p* value
	0–60	61–120	121–180	181–240	241–360	361–1440	
Crude in-hospital mortality among all patients, *n*/total (%), *n* = 1124	96/343 (28.0)	66/327 (20.2)	35/161 (21.7)	19/94 (20.2)	26/103 (25.2)	21/96 (21.9)	0.219
Crude in-hospital mortality among patients with septic shock, *n*/total (%), *n* = 703	68/215 (31.6)	47/207 (22.7)	27/94 (28.7)	16/61 (26.2)	21/69 (30.4)	16/57 (28.1)	0.466
Crude in-hospital mortality among patients from ED, *n*/total (%), *n* = 631	61/210 (29.1)	36/194 (18.6)	14/94 (14.9)	8/45 (17.8)	15/56 (26.8)	4/32 (12.5)	0.022
Adjusted in-hospital mortality among all patients (95% CI)^a^, *n* = 949	28.7 (23.3–34.1)*	21.6 (16.5–26.6)*	23.2 (16.4–30.0)	20.4 (12.1–28.7)	20.5 (12.6–28.5)	21.1 (12.5–29.7)	
Adjusted in-hospital mortality among patients with septic shock (95% CI)^a^, *n* = 615	30.0 (23.5–36.5)	22.3 (16.1–28.5)	28.5 (19.3–37.6)	27.4 (15.9–38.9)	25.1 (15.2–35.1)	21.6 (11.1–32.0)	
Adjusted in-hospital mortality among patients from ED (95% CI)^a^, *n* = 503	27.9 (21.1–34.6)*	18.4 (12.2–24.5)*	17.8 (9.3–26.2)	17.2 (5.4–28.9)	24.1 (12.2–36.0)	12.8 (5.7–25.0)	
Crude 28-day mortality, *n*/total (%), *n* = 1112	75/338 (22.2)	55/325 (16.9)	26/159 (16.4)	15/92 (16.3)	22/103 (21.4)	17/95 (17.9)	0.445
ICU-free days, median (IQR), *n* = 902	20 (12–24)	20 (13–24)	20 (9–23)	18 (6–24)	18 (10–24)	19 (1023)	0.3522
Ventilator-free days	21 (0–28)	21 (8–28)	22 (0–28)	20 (0–27)	21 (0–27)	20 (0–28)	0.3947
Length of hospital stay	26 (13–46)	22 (11–43)	26 (13–49)	21 (13–38)	25 (12–54)	29 (13–48)	0.4827

*ED* emergency department, *CI* confidence interval, *ICU* intensive care unit, *IQR* interquartile range

*< 0.05

^a^Adjusted by age, gender, admission source (emergency department, ward, or in intensive care unit), CCI, antibiotic use before arrival, site of infection (e.g., lung, abdomen, urinary tract, soft tissue, central nerve system, or blood stream-related), SOFA score, and intravenous fluid bolus completed within 3 h (30 mg/kg crystalloid)

## Discussion

### Summary

In emergency medical centers in Japan, one third of patients receive antibiotics within 1 h and three quarters within 3 h of sepsis recognition. Although we have found high levels of adherence to the sepsis care bundle (2012) during the study period, we were unable to show a linear relationship between the timing of antibiotic administration, such as within 1 h or 3 h after sepsis recognition, and in-hospital mortality among patients with severe sepsis and septic shock.

### Comparison with previous studies

Although previous studies have shown that a delay in antibiotic administration is associated with a higher in-hospital mortality rate [10–14], our findings from a prospective observational study obtained data of antibiotic administration timing do not support their results. Neither prospective nor retrospective observational data can establish causation, and discrepancies between these results only raise further questions. Interestingly, a meta-analysis of studies regarding the relationship between antibiotic administration timing and mortality found no significant benefit after administering antibiotics within 1–3 h from triage or sepsis recognition in comparison with administration at a later timing [15], and a RCT of providing antibiotics to patients with suspected sepsis in the ambulance did not show improved survival [7]. Nonetheless, these previous studies [7, 10–13] and the present study have three major problems: (1) understanding the clinical courses of sepsis, including its onset; (2) stratification of sepsis severity and cognate adjustments to treatment strategies, e.g., rapid treatments; and (3) establishing correlation or causation in sepsis care bundle studies.

Sepsis is different from other time-dependent and emergent conditions such as acute coronary syndrome because its precise onset is difficult to establish and the lack of a specific diagnostic marker makes recognition problematic. Three phases in the clinical course of sepsis are recognized: (A) time from onset of infection to a detectable condition, (B) time from a detectable condition to diagnosis, and (C) time from diagnosis to antibiotic administration (time to antibiotic), and although phases A and B are significantly shorter in other time-dependent emergent conditions, the duration of these two phases varies considerably in sepsis. Furthermore, it is difficult to focus only on phase C to reduce mortality in sepsis as other variables that can affect outcomes include the timestamp of antibiotic initiation (time zero) and patient location, as seen in previous studies [15] and in our study. Similar to other studies, we defined time zero as the time of sepsis recognition, although it is a subjective choice [15], because the clinical course of each patient would differ based on the presenting symptoms, even if triage at ED is chosen as a reliable time zero point [16]. Thus, detection of sepsis and antibiotic initiation are equally important as delays in appropriate antibiotic therapy are more likely to occur in patients with complex or atypical presentations who are also more likely to harbor drug-resistant organisms [17]. Moreover, reports show that inter-hospital transfer delayed the administration of initial antibiotics, although prognosis was not different [18]. Similar to previous reports, our patients included not only those presenting to ED but also those being transferred from other hospitals [15], and it is essential to disentangle such complicated definitions of time zero and locations in each study before direct comparisons can be made among these studies.

Sepsis severity stratification and adjustment with rapid treatment may have been insufficient in previous and our studies regarding time to antibiotic, and conflating sepsis and septic shock may also be a confounder. Although 63% of the patients in our study presented with shock, we could not find any association between earlier time to antibiotic and reduced mortality in all participants. Recent studies have demonstrated a weak association between time to antibiotic therapy and mortality in all cohorts (a relatively mild state in comparison with our cohort), although a large significant association between delays in antibiotic administration and higher mortality rates in patients with septic shock [10, 11]. Further, an RCT of administering antibiotics to patients with suspected sepsis in the ambulance also included many patients without shock [7]. In the study, it notes that even patients in the control group received antibiotics quite early and that the patients were treated with low risk (mortality rate 8%). This is quite a different population compared with that in the hospital setting. Indeed, severity adjustment is difficult in sepsis studies because sepsis includes complicated etiologies, various presentations, and severities compared with other medical emergencies [19]. Nevertheless, in our study, we used fewer variables to adjust severity in comparison with previous studies owing to the limitations of our database [12, 13]. Previous studies have reported higher crude mortality rates in patients administered with antibiotics in the first hour, which then declined over time; however, severity adjustment reversed the relationship between time to antibiotic and mortality [12, 13]. Our study may also have been under-adjusted for severity because the sample size was relatively smaller and covariates were fewer than those in previous studies [10–13].

Next, we must consider whether this relationship between time to antibiotic and mortality causative or only a correlation, given that all other components of the sepsis care bundle may be potentially physiologically effective along with time to antibiotic and that the effectiveness of each component remains controversial in various settings [10, 20, 21]. Adherence to the sepsis care bundle also varies in each setting [8, 10, 22, 23]. For example, in a study among patients who received antibiotics as they met the criteria for severe sepsis, a relatively high compliance rate for the sepsis care bundle was reported if sepsis was definitively diagnosed (with diagnosis code) compared with undiagnosed sepsis (without diagnosis code) [22]. In our study, the 0–60, 61–120, and 121–180 min groups were more likely to comply with the 3-h bundle, compared with the others. Conversely, the prognosis may have been good in patients who had been diagnosed early and in those who were administered with antibiotics early [10–13], implying that diagnosing sepsis may be more important than initiating the sepsis care bundle. Further, all components of the bundle should be investigated in future studies that focus on the relationship between time to antibiotic and trigger of initiation.

### Possible explanations and implications

Medical staff intuitively understands that the early use of appropriate antibiotics is an important modifiable factor. However, optimal timing and its effectiveness remain unclear after a widespread implementation of the sepsis protocol in developed countries such as Japan. Although there is no doubt that sepsis care bundle components, such as time to antibiotic, play a role in the arising importance of treatment speed, a similar situation arose when early goal-directed therapy was debated [24, 25].

In this study, patients who received antibiotics within the first hour had the highest mortality despite the greatest compliance with the entire 3-h bundle. This may be because of the severity at presentation, detecting antibiotic use within the first hour, or an obvious septic state. Alternatively, very early antibiotic use (within 1 h) may lead to unfavorable outcomes; although, this does not seem likely as all the patients would have had a sepsis state for some time before the point of recognition [26]. Furthermore, indiscriminate and rapid use of broad-spectrum antibiotics in all patients may be potentially harmful [27, 28], as it may lead to adverse events; however, there are only few reports on adverse events of rapid use of antibiotics. Incorrect diagnosis of sepsis may lead to a delay in administering other useful treatments or appropriate source control despite the use of broad-spectrum antibiotics [17].

Although there may be a linear relationship between early time to antibiotic therapy and better prognosis in sepsis, it is presumably weak. Moreover, similar to the previous study report, our results showed that earlier time to antibiotic therapy was related to better outcomes in ED patients [14] when we excluded patients who received antibiotics within 1 h. Otherwise, focusing on the rapid use of antibiotics may lead to misdiagnosis of the site of infection, which is related to poor outcomes [28]. Sepsis treatment and care involve components other than the time to antibiotic, and while this does not mean that rapid treatments are not good, most physicians intuitively recognize the need for it. Thus, timely antibiotic administration to patients with sepsis should remain one of the key elements in the sepsis care bundle, regardless of time restriction.

### Limitations

This study has several limitations. First, controlling for confounders may have been insufficient, as there are potential unknown confounders not available to us. Antibiotic initiation can be determined not only based on the patient’s severity but also on unknown factors that are difficult to be quantified. Second, there were patients who were using antibiotics before arrival. We have adjusted antibiotic use before arrival as a covariate in the GEE models. Moreover, the results did not change significantly even if patients with antibiotic usage before arrival were excluded. Third, there may be an indication bias as antibiotics may have been prescribed within the first hour only in patients with severe (non-survivable) presentation. However, we found that the results did not change even if patients who died within 1, 2, or 3 days were excluded from the analysis, suggesting that it may be difficult to show differences in practice quality between patients presenting with considerably mild and considerably severe symptoms [7]. Forth, there may have been a social desirability bias, as physicians usually wish to be evaluated as having responded to critical patients first. Fifth, there may have been a ceiling effect with respect to severity scores, because our study population was more severely ill than those in previous studies. Sixth, the timestamp of protocol initiation was defined as at the time of sepsis recognition, which is relatively later than the time of triage at ED. Our study may have only shown a relationship between time to antibiotic and in-hospital mortality if sepsis recognition occurred early in the course of the disease. Seventh, we did not have data about the appropriateness of antibiotic. However, as the first FORECAST paper [8] showed, 84% of patients received broad-spectrum antibiotics within 3 h. Most patients at least adhered to empiric antibiotics based on the guideline. In addition, carbapenem was most commonly used after initial diagnoses (55%) followed by tazobactam/piperacillin (21%) and vancomycin (18%). Thus, the important pathogens would have been covered by the chosen antibiotics even if antibiotic-resistant pathogens were present. Finally, the descriptive nature of the study could not completely identify the causal inference between the observed time to antibiotic and in-hospital mortality. In this study, we described sepsis care and implementation of sepsis bundle in Japan as one of the high adherence countries of the sepsis guideline. Our results suggested that this relationship is still controversial, and RCT of this topic should be studied further.

## Conclusions

Our prospective study failed to show a difference in in-hospital mortality based on the timing of antibiotic administration. Future studies are needed to prove or refute these results among patients with sepsis or septic shock.

## Data Availability

The datasets analyzed during the current study are available with the corresponding author on reasonable request.

## Abbreviations

ICU: Intensive care unit
FORECAST: Focused Outcomes Research in Emergency Care in Acute Respiratory Distress Syndrome, Sepsis, and Trauma
GEE: Generalized estimating equation model
SSC: Surviving Sepsis Campaign
RCT: Randomized controlled trial
JAAM: Japanese Association for Acute Medicine
VFD: Ventilator-free days
ED: Emergency department
SD: Standard deviation
IQR: Medians and interquartile range
CCI: Charlson comorbidity index
SOFA: Sepsis-related organ failure assessment
